# Systemic Inflammation Response Index Predicts Survival Outcomes in Glioblastoma Multiforme Patients Treated with Standard Stupp Protocol

**DOI:** 10.1155/2020/8628540

**Published:** 2020-11-14

**Authors:** Erkan Topkan, Ahmet Kucuk, Yurday Ozdemir, Huseyin Mertsoylu, Ali Ayberk Besen, Duygu Sezen, Yasemin Bolukbasi, Berrin Pehlivan, Ugur Selek

**Affiliations:** ^1^Baskent University Medical Faculty, Department of Radiation Oncology, Adana, Turkey; ^2^Mersin City Hospital, Radiation Oncology Clinics, Mersin, Turkey; ^3^Baskent University Medical Faculty, Department of Medical Oncology, Adana, Turkey; ^4^Koc University, School of Medicine, Department of Radiation Oncology, Istanbul, Turkey; ^5^The University of Texas, MD Anderson Cancer Center, Department of Radiation Oncology, Houston, TX, USA; ^6^Department of Radiation Oncology, Bahcesehir University, Istanbul, Turkey

## Abstract

**Objectives:**

We endeavored to retrospectively assess the prognostic merit of pretreatment systemic immune response index (SIRI) in glioblastoma multiforme (GBM) patients who underwent postoperative partial brain radiotherapy (RT) and concurrent plus adjuvant temozolomide (TMZ), namely, the Stupp protocol.

**Methods:**

The records of 181 newly diagnosed GBM patients who received the postoperative Stupp protocol were retrospectively analyzed. The SIRI value for each eligible patient was calculated by utilizing the platelet, neutrophil, and lymphocyte measures obtained on the first day of treatment: SIRI = Neutrophils × Monocytes/Lymphocytes. The ideal cutoff values for SIRI connected with the progression-free- (PFS) and overall survival (OS) results were methodically searched through using the receiver operating characteristic (ROC) curve analysis. Primary and secondary end-points constituted the potential OS and PFS distinctions among the SIRI groups, respectively.

**Results:**

The ROC curve analysis labeled the ideal SIRI cutoffs at 1.74 (Area under the curve (AUC): 74.9%; sensitivity: 74.2%; specificity: 71.4%) and 1.78 (AUC: 73.6%; sensitivity: 73.1%; specificity: 70.8%) for PFS and OS status, individually. The SIRI cutoff of 1.78 of the OS status was chosen as the common cutoff for the stratification of the study population (Group 1: SIRI ≤ 1.78 (*N* = 96) and SIRI > 1.78 (*N* = 85)) and further comparative PFS and OS analyses. Comparisons between the two SIRI cohorts manifested that the SIRI ≤ 1.78 cohort had altogether significantly superior median PFS (16.2 versus 6.6 months; *P* < 0.001) and OS (22.9 versus 12.2 months; *P* < 0.001) than its SIRI > 1.78 counterparts. The results of multivariate Cox regression analyses ratified the independent and significant alliance between a low SIRI and longer PFS (*P* < 0.001) and OS (*P* < 0.001) durations, respectively.

**Conclusions:**

Present results firmly counseled the pretreatment SIRI as a novel, sound, and independent predictor of survival outcomes in newly diagnosed GBM patients intended to undergo postoperative Stupp protocol.

## 1. Introduction

The contemporary standard of care for glioblastoma multiforme (GBM) incorporates maximum tumor resection pursued by radiotherapy (RT) and concurrent plus maintenance temozolomide (TMZ), namely, the Stupp protocol [1]. However, despite the critical advancements in diagnostic tools and surgical techniques over the most recent two decades, no conspicuous enhancements have been accomplished in survival results starkly contrasted with other gliomas, with a 5-year survival estimate of only 9.8% in the most optimistic case scenario [2]. Additionally, neither the adjustments in RT nor chemotherapy schedules could scientifically prove marked gains in survival outcomes of such patients past that typically attained with the standard Stupp convention [3–5].

The well-established prognostic factors for GBM comprise the patients' age, neurologic function status, Karnofsky performance status (KPS), recursive partitioning analysis (RPA) group, the extent of curative resection, concurrent plus maintenance TMZ administration status, and the presence/absence of the genetic and molecular markers, like O6-methylguanine-DNA methyl-transferase (MGMT) gene promoter methylation, isocitrate dehydrogenase 1/2 (IDH-1/2) mutation, and 1p/19q codeletion [6, 7]. These key factors assuredly yield valuable information about the overall prognosis of GBM patients. Nevertheless, the significant disparities among the ultimate outcomes of comparable patients in terms of clinical, pathological, genetic, and molecular properties after the Stupp protocol stress the essential prerequisite for the identification of novel prognosticators with superior discriminatory strength to better laminate the patients for individualized treatment maneuvers.

Growing proof suggests the host immunity and inflammation as the two firmly related conditions that impact glioma development and progression [7, 8]. Various blood-born host biomarkers of immunity and inflammation have been studied for their predictive and prognostic powers in gliomas, including the GBM: C-reactive protein, albumin, platelets, monocytes, neutrophils, lymphocytes, neutrophil to lymphocyte ratio (NLR), platelet to lymphocyte ratio (PLR), lymphocyte to monocyte ratio (LMR), prognostic nutritional index (PNI), Glasgow prognostic score (GPS), and lately, the systemic immune inflammation index (SII) and C-reactive protein to albumin ratio [9–16]. The consistent results of these independent investigations ascertained that each biomarker or their distinctive blends were robustly associated with the clinical outcomes of the GBM patients. Latterly, Qi et al. exhibited that the novel systemic inflammation response index (SIRI), which combines the circulating neutrophils, monocytes, and lymphocytes, was firmly associated with the outcomes of pancreatic cancer patients following systemic chemotherapy [17]. Consequent separate reports on esophageal squamous cell-, nasopharyngeal-, hepatocellular-, and renal clear cell carcinomas additionally alleged the independent prognostic value of SIRI at these tumor sites, as well as [18–21]. Nevertheless, diverging from its prognostic potential, to our best understanding, the significance of SIRI in the forecast of clinical outcomes of GBM patients has never been addressed before. Therefore, we conducted this retrospective cohort analysis to uncover the potential prognostic utility of SIRI in newly diagnosed GBM patients who underwent the standard Stupp protocol.

## 2. Patients and Methods

### 2.1. Study Cohort

The treatment charts of all newly diagnosed GBM patients who underwent the postneurosurgical partial brain RT with concurrent TMZ and up to 6-12 cycles of adjuvant TMZ between February 2007 and December 2017 were retrospectively examined. Patients satisfying the following criteria were deemed eligible: (1) aged 18 to 80 years, (2) Karnofsky performance score (KPS) ≥ 70, (3) histologically proved GBM diagnosis, (4) no past cranial RT and/or chemotherapy history, (5) available pre- and postoperative gadolinium-enhanced magnetic resonance imaging (MRI) scans, (6) present pretreatment complete blood count and chemistry tests with sufficient hematologic, renal, and hepatic functions, (8) no direct evidence for active infection, and (9) no history of immunosuppressive therapy for any reason.

### 2.2. Ethics, Consent, and Permissions

The present study protocol was sketched granting to the postulates of the Declaration of Helsinki and its amendments and was approved by the institutional review council before the gathering of any patient information. Each qualified patient provided signed informed consent before the commencement of treatment either themselves or lawfully commissioned deputies for compilation and interpretation of blood samples, pathologic specimens, and academic publication of their results.

### 2.3. Treatment Protocol

As designated by our institutional standards for GBMs, all patients were first evaluated for maximal safe neurosurgical resection and underwent this procedure if elected feasible. Postoperative 3-dimensional conformal- or simultaneous integrated boost intensity-modulated RT to a total dose of 60-70 Gy (2.0 or 2.33 Gy/fx) over 6 weeks was delivered, as displayed in detail, elsewhere. All patients received TMZ (75 mg/m2/day once daily, 7-days/week) and prophylactic trimethoprim-sulfamethoxazole against Pneumocystis jirovecii during the entire course of concurrent RT and TMZ. Adjuvant TMZ was prescribed one month after the completion of RT chemotherapy comprised maintenance (150/200 mg/m2/day, for 5-days, every 28 days) up to 6-12 cycles.

### 2.4. Measurement of SIRI

The SIRI was calculated as follows by using the Qi's original SIRI formula [17]: *N* × *M*/*L*, where *N*, *M*, and *L* indicate the pretreatment neutrophil, monocyte, and lymphocyte counts obtained on the first day of treatment, respectively.

### 2.5. Response Assessment

Treatment response according to the Response Assessment in Neuro-Oncology (RANO) working group report was assessed utilizing the brain MRI every 2 months for the first- and every 3 months for the second follow-up years and every 6 months or more frequently if necessitated, thereafter [22]. While the de novo stipulation for the initiation or increment of the already prescribed daily corticosteroid dose was accepted and registered as a symptomatically progressive disease if brain MRI could not be performed.

### 2.6. Statistical Analysis

The primary and secondary intentions of this retrospective cohort examination were the likely influence of the SIRI levels on overall- (OS: the interim between the first day of the concurrent RT and TMZ and the date of death/the last visit) and progression-free survival (PFS: the interim between the first day of the RT and TMZ and first recorded date of disease progression or death/last visit) results, individually. Chi-square or Fisher's exact tests were used to analyze the quantitative- (measured with medians and ranges) and categorical variables (measured with frequencies and percentages), respectively. The accessibility of an ideal SIRI cutoff that unveils significant interplay with the PFS and OS results was sought by utilizing the receiver operating characteristic (ROC) curve analysis. The demographic features of SIRI groups were compared with performing the Pearson *χ*^2^ test. Pearson's exact test or Spearman's correlation analyses were utilized to correlate any two factors as relevant. The Kaplan-Meier survival curves and two-sided Log-rank tests were performed for survival analyses and intergroup comparisons. Cox Proportional Hazard model was utilized for the multivariate analyses to assess the potential links between the variables with univariate significance and survival outcomes. All comparisons were 2-tailed, and any *P* < 0.05 was valued as significant.

## 3. Results

### 3.1. Patient Demographics

The retrospective search of the Baskent University Medical Faculty, Department of Radiation Oncology revealed a sum of 252 consecutively treated GBM patients. However, leaving 181 patients qualified for this investigation, 71 of them were assessed unfit due to receiving hypofractionated short-course RT (*n* = 38), chronic use of immunosuppressive agents (*n* = 14), active viral or bacterial infection (*n* = 7), reluctance for receiving concurrent (*n* = 7), and adjuvant TMZ (*n* = 5), respectively. Baseline patient and disease characteristics for the whole investigation accomplice were as summarized in Table 1. The median age was 59 years (range: 24–80 years), with males (65.9%) constituting the majority of the patients. Steroids and anticonvulsants were noted to be already prescribed in 65.7% and 37% of the patients before the admission period, respectively. Median symptom duration was 2.3 months (range: 0.2 - 7.9 months), with 71.3% cases being diagnosed within 3 months of the first manifestation of the symptom(s). Gross total excision (GTR) was performed in 37.0% cases, while subtotal excision (STR) was the most frequent neurosurgical intervention (48.4%) to be practiced. As a direct result of the accessibility of these specific tests after 2016 in our country, the IDH1/2 mutation status was available in 93 (10.8) cases, and just 10 (10.8%) of them were tested positive.

### 3.2. Ideal SIRI Cutoff Value

**Figure 1:**
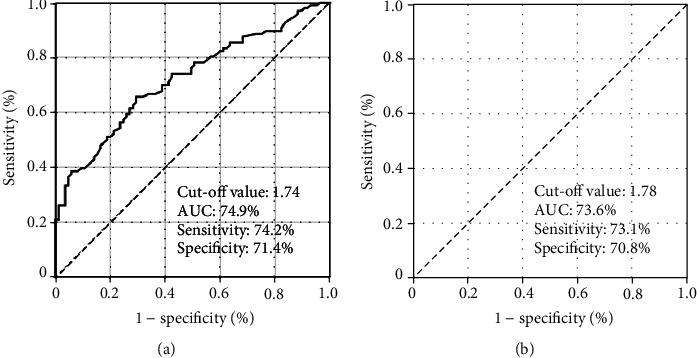
Results of the receiver-operating characteristic curve analysis for disease-free- and overall survival status.

In order to reveal the presence of ideal cutoffs for likely reciprocities between the SIRI values and PFS and OS status, we employed ROC curve analysis as a broadly appreciated objective statistical method for such analyses. As sketched in Figure 1, the ideal cutoff values were distinguished as 1.74 (Area under the curve (AUC): 74.9%; sensitivity: 74.2%; specificity: 71.4%) and 1.78 (AUC: 73.6%; sensitivity: 73.1%; specificity: 70.8%) for PFS and OS status, separately. Nevertheless, the 1.78 value of the OS status was chosen as the common cutoff to laminate the patients into two distinctive cohorts for further comparative PFS and OS analyses, as the two cutoffs were very close to each other. Consequently, the patients' cohort was dichotomized into SIRI ≤ 1.78 (*N* = 96) and SIRI > 1.78 (*N* = 85) gatherings, respectively. Except for a higher requirement for hospitalization due to the acute treatment-related complications during the concurrent RT and TMZ period (10.4% versus 22.4%; *P* = 0.002) and an associated longer hospitalization duration (4 days versus 9 days (*P* < 0.001) in the SIRI > 1.78 cohort, we could not demonstrate any remarkable discrepancies between the two SIRI groups in terms of baseline demographics (Table 1) or upfront and salvage treatment attributes (Table 2).

### 3.3. Recurrence Patterns and Salvage Treatment

At a median follow-up of 15.9 months (range: 1.0 - 108.7 months), 17 (9.3%) of them were still alive, and 15 (8.2%) of them were free of disease progression during this final analysis. As exhibited in Table 3, all 166 relapses were reported to be intracranial disease progression with no additional extracranial metastasis. Intracranial progression rate was significantly higher in the SIRI > 1.78 group (95.3% versus 88.5% for SIRI ≤ 1.78; *P* = 0.0014). Overall, the infield and marginal relapses cumulatively accounted for 85.1% of all relapses with no significant differences between the two SIRI groups. Salvage treatments specified in Table 2 were administered to 98 (58.1%) relapsed patients, and there were no statistically meaningful discrepancies between the two SIRI groups in both terms of salvage treatment frequencies and the specific rescue intervention(s).

### 3.4. Impact of SIRI Values on Survival Outcomes

**Figure 2:**
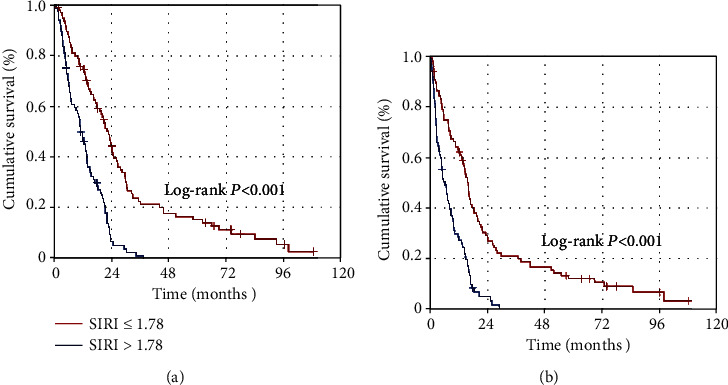
Comparative results of the progression-free- and overall survival outcomes per systemic immune response index (SIRI) cohorts: SIRI ≤ 1.78 (red line) and >0.75 (dark blue line).

For the whole research cohort, the median, 2-year, and 5-year PFS estimates were 10.3 months (95% confidence interval (CI): 7.2-13.4 months), 11.9%, and 6.6%, respectively, while the corresponding OS estimates were 15.8 months (95% CI: 12.5-19.1 months), 27.2%, and 8.2%, individually. Comparative analyses between the two SIRI cohorts exhibited that both the median PFS (16.2 (95% CI: 14.5-17.9 months) versus 6.6 months (95% CI: 3.1-8.9 months)) and OS (22.9 (95% CI: 19.8–26.0 months) versus 12.2 (95% CI: 9.7–14.6 months)) durations were significantly superior in the SIRI ≤ 1.78 cohort than its SIRI > 1.78 counterpart (Figure 2). Additionally, the 5-year PFS (11.9% versus 0%) and OS (15.3% versus 0%) estimates were likewise numerically superior in the SIRI ≤ 1.78 cohort, as outlined in Table 2.

### 3.5. Univariate and Multivariate Outcomes

As displayed in Table 4, the results of univariate investigation exhibited that the KPS 90-100 versus 70-80 (*P* = 0.002 for PFS and *P* = 0.001 for OS), RTOG RPA classes III-IV versus V (*P* < 0.001 for PFS and OS), gross total resection versus subtotal resection/biopsy only (*P* = 0.006 for PFS and *P* = 0.009 for OS), IDH-mutated versus wild type (*P* < 0.001 for PFS and OS), and the SIRI ≤ 1.78 versus SIRI > 1.78 (*P* < 0.001 for PFS and OS) were the confounders significantly linking with the respective PFS and OS results (Table 4). The results of the multivariate analyses firmly demonstrated the independent significance of the association among the abovementioned five factors and the PFS and OS outcomes: *P* < 0.05 for the link between the each variable and each survival endpoint (Table 4).

## 4. Discussion

Consequences of this first report investigating the prognostic efficiency of the pretreatment SIRI in GBM patients unsealed that a high SIRI level was linked to significantly reduced median, and long-term PFS and OS result in newly diagnosed GBM patients managed with the standard Stupp protocol. Therefore, other than exposing the prognostic worth of the SIRI in such patients, our outcomes further suggested that pretreatment immune and inflammatory marker SIRI may serve independently useful in more isolated stratification of the GBM patients into two exclusive gatherings with noticeably distinct survival forecasts.

The undisputed robust interconnection between the systemic inflammation and virtually all steps of carcinogenesis and its gradual progression led to the widespread ratification of the inflammation as the seventh hallmark of cancer [23]. Cellular inflammatory constituents are omnipresent in the microenvironment of the numerous cancer tissues, including the GBM [23]. For GBM, these constituents involve the eosinophils, neutrophils, monocytes, B- and T-lymphocytes, tumor-associated macrophages, myeloid-derived suppressor cells, dendritic cells, and M1 and M2 microglia [24]. Because these immune cells carry out fundamental functions in gliomagenesis induction and progression through secretion of plentiful proinflammatory chemokines, cytokines, and growth factors promoting the tumor proliferation, invasiveness, metastasis, angiogenesis, antitumor immunity suppression, and resultant facilitated escape from the immune system [24]. Consequently, the aggravated and uncontrolled local and systemic inflammatory responses have been extensively studied and attested to be firmly correlated with the prognosis of cancer patients independent of the conservative tumor-related prognosticators [23]. As such, various peculiar blends of immune and inflammatory markers have been searched for their prognostic efficiency in GBM patients, in like manner their counterparts presenting with extracranial cancers: Neutrophils, lymphocytes, platelets, monocytes, NLR, PLR, C-reactive protein, albumin, PNI, GPS, SII, and CRP/Alb with results demonstrating strong and independent prognostic values for each parameter, separately [7–16]. Establishing wise bases for our current research, Qi's SIRI has never been considered before for its likely prognostic usefulness in GBM patients, regardless of the truth convincingly demonstrating its vital competence in prognostic stratification of patients manifesting with various extracranial cancers [17–21].

Other than sanctioning the prognostic usefulness of the universally appreciated KPS, RTOG RPA class, extent of the surgery, and IDH1/2 mutation status, the principal finding of the present investigation was the remarkable exhibit of a compelling and independent prognostic worthiness for the baseline SIRI at a cutoff value of 1.78 in newly diagnosed GBM patients who underwent the standard Stupp protocol. Our current outcomes, which represent the direct results of the first academic endeavor rigorously evaluating the prognostic efficiency of SIRI in such patients aggregate, clearly demonstrated that the SIRI ≤ 1.78 values were associated with significantly superior median PFS (16.2 versus 10.3 months; *P* < 0.001) and OS (22.9 versus 12.2 months; *P* < 0.001) outcomes opposed to their SIRI > 1.78 counterparts. Furthermore, the corresponding superior 5-year PFS (11.9% versus 0%) and OS (15.3% versus 0%) rates conjointly appeared to propose long-term durability of the prognostic significance of the low pretreatment SIRI values after the standard Stupp protocol. In like manner, the notable absence of 5-year survivors in the high SIRI accomplice also suggested an extremely aggressive, rapidly relapsing, resistant to salvage treatment(s), and inevitably fatal GBM phenotype regardless of the use of identical salvage interventions in both groups. Robustly confirming this sensible suggestion, an overall 4.7% brain-failure rate observed in the SIRI > 1.78 patients' group was significantly lower than the 11.5% rate observed in the SIRI ≤ 1.78 group (*P* = 0.014). Though it is strenuous to discuss our results in an evidence-based manner in lack of published GBM-specific SIRI research results, yet they are in good agreement with the outcomes of previously published SIRI literature for other cancer sites [17–21] and a SII study in GBM patients [16]. For a notable example, the results of our recently reported research investigating the influence of SII in a similarly managed GBM cohort of 167 patients indicated meaningfully extended median PFS (16.6 versus 6.0 months; *P* < 0.001) and OS (22.9 versus 11.1 months; *P* < 0.001) durations in the cohort with SII < 565 those reported for the SII ≥ 565 after the same treatment protocol utilized here [16]. Considering the apparent similarities between the previous SII (*P* × *N*/*L*) and present SIRI (*N* × *M*/*L*) formulas, where just the platelets of the SII has been supplanted by the monocytes in the SIRI, the congruency among the two immune response indexes is not surprising, as both cell types have been sufficiently established to enhance tumor growth, immune escape, and cell survival [24].

The precise mechanisms underlying the alliance between high SIRI levels and poor survival results prevail to be uncovered. However, still, some sensible comments can be inferred on this particular issue of supreme importance by thoroughly considering the well-established crucial roles of local and systemic inflammation in all means of the gliomagenesis initiation and its malignant progression together with the inflammatory cell ingredients of the unique SIRI formula: monocytes, neutrophils, and lymphocytes. The microenvironment of gliomas, particularly the GBM, is profoundly inflammatory and immunosuppressive, which expedites the evasion of glioma cells from the immune system, namely, the cardinal antitumoral defense framework [25]. Monocytes and their highly specialized forms, macrophages, play major roles in cancer initiation and progression by exerting crucial actions on the tumor cell migration, invasion, intravasation, metastasis, tumor-associated angiogenesis, and suppression of antitumor immune reaction [25–27]. As a source of tumor-associated macrophages, monocytes are recruited to the brain parenchyma in any pathological condition which is particularly relevant to gliomas [28–30]. For GBMs, in a previous in vitro study, it has been clearly demonstrated that the normal human monocytes were acquiring the tumor-promoting immunosuppressive features of myeloid-derived suppressor cells upon exposure to GBM cells via cell-to-cell contact [31]. In this respect, reflecting a poor antitumor immune status, the results of a meta-analysis incorporating 56 studies and 20,248 patients with various cancers demonstrated that a low lymphocyte to monocyte ratio, or reversely a high monocyte to lymphocyte ratio, was a strong indicator of significantly poor survival outcomes, which was associated with clinicopathological features of aggressive disease phenotype [32]. GBMs have been shown to exhibit the highest neutrophil infiltration among all gliomas [33], with increased neutrophils recruitment being associated with promoted tumor progression and resistance to treatment [34]. Additionally, creating an immunosuppressive milieu, neutrophils may inhibit cytolytic CD8^+^ T-cells and natural killer cells and may suppress CD4^+^ suppressor T cells, which may facilitate the GBM cell survival and disease progression [35]. Lending support to such evidence, Han et al. demonstrated a link between the increased neutrophil infiltration levels and diminished survival times in GBM patients [36]. In contrary to the monocytes and neutrophils, lymphocytes behave as the key components of the antitumor immunity via exerting direct and antigen-dependent cytotoxic cell death functions and suppression of the tumoral proliferation, growth, and invasion [37]. Furthermore, lengthened survival durations were shown to be closely and directly associated with the increased levels of tumor-infiltrating lymphocytes [38]. Therefore, although the underlying mechanisms might be more complex, yet, as we witnessed herein, the cumulative influence of the decreased levels of the immunogenic lymphocytes and the increased levels of the immunosuppressive monocytes and neutrophils might be responsible to some extent for the deteriorated PFS and OS results in GBM patients presenting with SIRI > 1.78.

The present investigation demonstrates several potential weaknesses. First, the results exhibited here need to be certified with the outcomes of competently planned ensuing research treating larger patient populations as they represent the findings of a single institutional small-scale retrospective cohort survey with unforeseeable potential biases of such kind of studies. Second, the noticeable absence of correlative analyses between the genomic markers including the MGMT methylation and isocitrate dehydrogenase-1/2 (IDH1/2) mutation status and the SIRI groups constrained our capacity to conclude on the possibly significant associations between these factors. Third, the neutrophil, monocyte, and lymphocyte quantities may exhibit broad variations during the RT plus TMZ and maintenance TMZ periods. Nevertheless, our SIRI measures indicated just a single time point values. Upcoming studies measuring the SIRI at multiple time points during the concurrent and maintenance TMZ periods may serve valuably in more accurate determination of the perfect SIRI cutoff value for GBM patients, which may stratify such patients into more distinct prognostic groups. And fourth, the differences between the salvage procedures may moreover have unpredictably adjusted the results in favor of one SIRI group. For these reasons, present results ought to be interpreted with caution and valued as hypothesis-generating until the results of properly designed additional large-scale studies become accessible.

## 5. Conclusions

The outcomes of our present retrospective cohort analysis in 187 newly diagnosed GBM patients indicated that a high pretreatment SIRI measure was unequivocally connected with significantly deteriorated survival outcomes after the standard Stupp regime independent of the well-established prognostic factors in this patients gathering. Consequently, if validated with competently designed separate investigations, the simple to achieve and calculate immune and inflammation marker SIRI might be dependably utilized in further prognostic lamination and wise selection of the most convenient treatment option for the GBM patients on a per-patient basis.

## Figures and Tables

**Table 1:** Baseline demographics per SIRI status.

Characteristic	All patients (*n* = 181)	SIRI ≤ 1.78 (*n* = 96)	SIRI > 0.78 (*n* = 85)	*P* value
Median age, y (range)	59 (24-80)	60 (32-79)	58 (24-80)	0.54
Age group, *n* (%)				
<50 years	57 (31.5)	30 (31.3)	27 (31.8)	0.88
≥50 years	124 (68.5)	66 (68.7)	58 (68.2)	
Gender, *n* (%)				
Female	65 (35.9)	34 (35.4)	31 (36.5)	0.84
Male	116 (64.1)	62 (64.6)	54 (63.5)	
KPS, *n* (%)				
90-100	98 (54.1)	55 (57.3)	43 (50.6)	0.17
70-80	83 (45.9)	41 (42.7)	42 (49.4)	
Presenting symptoms				
Focal sensory-motor deficit	96 (53.0)	49 (51.0)	47 (55.3)	0.49
Cognitive deficits	44 (24.3)	23 (24.0)	21 (24.7)	
Increased ICP	31 (17.1)	19 (19.8)	12 (14.1)	
Others	10 (5.6)	5 (5.2)	5 (5.9)	
Symptom duration, *n* (%)				
<3 months	129 (71.3)	71 (74.0)	58 (68.2)	0.28
≥3 months	52 (28.7)	25 (26.0)	27 (31.8)	
Corticosteroid use, *n* (%)				
Yes	119 (65.7)	59 (61.5)	60 (70.6)	0.08
No	62 (34.3)	37 (38.5)	25 (29.4)	
Anticonvulsant use, *n* (%)				
Yes	67 (37.0)	35 (36.5)	32 (37.6)	0.90
No	114 (63.0)	61 (63.5)	53 (62.4)	
Tumor location, *n* (%)				
Frontal	39 (21.5)	20 (20.8)	19 (22.4)	0.53
Parietal	32 (17.8)	15 (15.7)	17 (20.0)	
Temporal	37 (20.4)	20 (20.8)	17 (20.0)	
Occipital	19 (10.5)	11 (11.5)	8 (9.4)	
Midline	19 (10.5)	10 (10.4)	9 (10.5)	
Multilobar	35 (19.3)	20 (20.8)	15 (17.7)	
Extent of surgery, *n* (%)				
GTR	67 (37.0)	37 (38.5)	30 (35.3)	0.66
STR	79 (43.7)	42 (43.8)	37 (43.5)	
Biopsy	35 (19.3)	17 (17.7)	18 (21.2)	
IDH status^∗^				
Positive	10 (10.8)	5 (10.4)	5 (11.1)	0.78
Negative	83 (89.2)	43 (89.6)	40 (88.9)	
RTOG RPA class, *n* (%)				
III	69 (38.1)	37 (38.5)	32 (37.6)	0.74
IV	77 (42.5)	42 (43.3)	35 (41.2)	
V	35 (19.4)	17 (18.2)	18 (21.2)	

^∗^IDH status was available in 93 patients. Abbreviations: SIRI: systemic immune response index; KPS: Karnofsky performance score; ICP: intracranial pressure; GTR: gross total resection; STR: subtotal resection; IDH: isocitrate dehydrogenase; RTOG RPA: radiation therapy oncology group recursive partitioning analysis.

**Table 2:** Treatment characteristics and clinical outcomes.

Characteristic	All patients (*n* = 181)	SIRI ≤ 1.78 (*n* = 96)	SIRI > 1.78 (*n* = 85)	*P* value
RT technique, *n* (%)				
3D-CRT	94 (51.9)	49 (51.0)	45 (52.9)	0.79
SIB-IMRT	87 (48.1)	47 (49.0)	40 (47.1)	
RT dose, *n* (%)				
60 Gy	97 (53.6)	50 (52.1)	47 (55.3)	0.62
70 Gy	84 (46.4)	46 (47.9)	38 (44.7)	
Adjuvant TMZ cycles, *n* (%)				
1-5	48 (28.8)	23 (28.8)	25 (28.7)	0.96
6-12	119 (71.2)	57 (71.2)	62 (71.3)	
Salvage treatment, *n* (%)				
None	83 (45.9)	44 (45.8)	39 (45.9)	0.54
Unknown	7 (3.9)	4 (4..2)	3 (3.5)	
SNS alone	19 (10.5)	10 (10.4)	9 (10.7)	
SRS/SRT	16 (8.8)	8 (8.4)	8 (9.4)	
SNS + SRS/SRT	8 (4.4)	5(5.2)	3 (3.5)	
SNS + Ctx	15 (9.3)	8 (8.4)	7 (8.2)	
SNS + SRS + Ctx	8 (4.4)	4 (4.2)	4 (4.7)	
Ctx alone	25 (12.8)	13 (13.4)	12 (14.1)	
Need for hospitalization, **n** (%)				
Yes	29 (16.0)	10 (10.4)	19 (22.4)	0.002
No	152 (84.0)	86 (89.6)	66 (77.6)	
Hospitalization duration, days	6 (1-17)	4 (1-8)	9 (3-17)	< 0.001

Abbreviations: SIRI: systemic immune response index; RT: radiotherapy; 3D-CRT: 3-dimensional conformal radiotherapy; SIB-MRT: simultaneous integrated boost intensity-modulated radiotherapy; TMZ: temozolomide; SNS: salvage neurosurgery; SRS: stereotactic radiosurgery; SRT: stereotactic radiotherapy; Ctx: chemotherapy.

**Table 3:** Brain failure and survival outcomes per SIRI group.

Characteristic	All patients (*N* = 181)	SIRI < 1.73 (*N* = 96)	SIRI > 1.73 (*N* = 85)	*P* value
Brain failure, *n* (%)				
Present	15 (8.2)	11 (11.5)	4 (4.7)	0.014
Absent	166 (91.8)	85 (88.5)	81 (95.3)	
Brain failure pattern, *n* (%)				
None	15 (8.2)	11 (11.5)	4 (4.7)	0.53
Infield	140 (77.4)	73 (76.0)	67 (78.7)	
Marginal	14 (7.7)	4 (4.2)	10 (11.8)	
Distant	5 (2.8)	3 (3.1)	2 (2.4)	
Infield and distant	4 (2.2)	3(3.1)	1 (1.2)	
Marginal and distant	3 (1.7)	2 (2.1)	1 (1.2)	
PFS				
Median, mo (95% CI)	10.3 (7.2-13.4)	16.2 (14.5-17.9)	6.6 (3.1-8.9)	<0.001
2 years (%)	11.9	29.5	5.0	
5 years (%)	6.6	11.9	0	
OS				
Median, mo (95% CI)	15.8 (12.5-19.1)	22.9 (19.8-26.0)	12.2 (9.7-14.6)	<0.001
2 years, %	27.2	44.6	5.5	
5 years, %	8.2	15.3	0	

Abbreviations: SIRI: systemic immune response index; CI: confidence interval; PFS: progression-free survival; OS: overall survival.

**Table 4:** Results of uni- and multivariate analysis.

Variable	PFS			OS		
	Univariate *P* value	Multivariate *P* value	Hazard ratio	Univariate *P* value	Multivariate *P* value	Hazard ratio
Age (≤50 vs. >50 years)	0.17	—	—	0.14	—	—
Gender (male vs. female)	0.84	—	—	0.92	—	—
KPS (90-100 vs. 70-80)	0.002	0.008	1.48	0.001	0.005	1.57
RTOG RPA group (III-IV vs. V)	<0.001	<0.001	1.98	<0.001	<0.001	2.14
Symptom duration (<3 vs. ≥3 months)	0.42	—	—	054	—	—
Extent of resection (GTR vs. STR/biopsy)	0.006	0.014	1.72	0.009	0.019	1.68
IDH status (positive vs. negative)	<0.001	<0.001	2.33	<0.001	<0.001	2.94
RT technique (3D-CRT vs. SIB-IMRT)	0.91	—	—	0.94	—	—
RT dose (60 vs. 70 Gy)	0.43	—	—	0.55	—	—
SIRI group (<1.78 vs. ≥1.78)	<0.001	<0.001	2.07	<0.001	<0.001	2.77

Abbreviations: PFS: progression-free survival; OS: overall survival; KPS: Karnofsky performance score; RTOG-RPA: radiation therapy oncology group recursive partitioning analysis; GTR: gross total resection; STR: subtotal resection; IDH: isocitrate dehydrogenase; 3D-CRT: 3-dimensional conformal radiotherapy; SIB-IMRT: simultaneous integrated boost intensity-modulated radiotherapy; RT: radiotherapy; SII: systemic immune-inflammation index; SIRI: systemic immune response index.

## Data Availability

The datasets used and/or analyzed during the current study are available from the Baskent University Department of Radiation Oncology Institutional Data Access for researchers who meet the criteria for access to confidential data: contact address: adanabaskent@baskent.edu.tr.
